# Molecular Confirmation of *Rickettsia parkeri* in *Amblyomma ovale* Ticks, Veracruz, Mexico

**DOI:** 10.3201/eid2512.190964

**Published:** 2019-12

**Authors:** Sokani Sánchez-Montes, Gerardo G. Ballados-González, Alejandra Hernández-Velasco, Héctor M. Zazueta-Islas, Marlene Solis-Cortés, Haydee Miranda-Ortiz, Julio C. Canseco-Méndez, Edith A. Fernández-Figueroa, Pablo Colunga-Salas, Andrés M. López-Pérez, Jesús Delgado-de la Mora, Jesús D. Licona-Enriquez, David Delgado-de la Mora, Sandor E. Karpathy, Christopher D. Paddock, Claudia Rangel-Escareño

**Affiliations:** Universidad Nacional Autónoma de México, Mexico City, Mexico (S. Sánchez-Montes, H.M. Zazueta-Islas, M. Solis-Cortés, E.A. Fernández-Figueroa, P. Colunga-Salas, A.M. López-Pérez);; Universidad Veracruzana, Veracruz, Mexico (G.G. Ballados-González, A. Hernández-Velasco);; Instituto Nacional de Medicina Genómica, Mexico City (H. Miranda-Ortiz, J.C. Canseco-Méndez, E.A. Fernández-Figueroa, C. Rangel-Escareño);; University of California, Davis, California, USA (A.M. López-Pérez);; Instituto Nacional de Ciencias Médicas y Nutrición Salvador Zubirán, Mexico City (J. Delgado-de la Mora);; Centro Médico Nacional Siglo XXI, Mexico City (J.D. Licona-Enriquez);; Instituto Tecnológico de Sonora, Sonora, Mexico (D. Delgado-de la Mora);; Centers for Disease Control and Prevention, Atlanta, Georgia, USA (S.E. Karpathy, C.D. Paddock)

**Keywords:** dogs, sentinel, rickettsiosis, emerging pathogen, *Rickettsia parkeri*, *Amblyomma ovale*, *gltA*, *htrA*, *sca0*, *sca5*, Atlantic Rainforest strain, ticks, tickborne disease, vector-borne infections, Veracruz, Mexico, bacteria, molecular characterization

## Abstract

We found *Rickettsia parkeri* in *Amblyomma ovale* ticks collected in Veracruz, Mexico, in 2018. We sequenced gene segments of *gltA*, *htrA*, *sca0*, and *sca5*; phylogenetic reconstruction revealed near-complete identity with *R*. *parkeri* strain Atlantic Rainforest. Enhanced surveillance is needed in Mexico to determine the public health relevance of this bacterium.

*Amblyomma ovale* hard ticks are located predominantly in South and Central America but can also be found in areas of the nearctic, particularly Mexico and the southern United States (*1*,*2*). Immature stages of this species parasitize many mammal and bird species, and adults complete their life cycle on artiodactyls and carnivores, particularly canids (*1*,*3*). *A*. *ovale* ticks have been collected predominantly in sylvatic areas, but because free-roaming dogs often enter sylvatic habitats and return to peridomestic settings with attached ticks, these ticks have become distributed into transitional and rural environments (*3*). In Brazil, this species has been implicated as the main vector of the *Rickettsia parkeri* strain Atlantic Rainforest, an eschar-associated spotted fever pathogen (*3*,*4*). Since its discovery, strain Atlantic Rainforest has been detected in other hard tick species, including *A*. *aureolatum* and *Rhipicephalus sanguineus* sensu lato in Argentina, Colombia, and Belize (*4*–*6*).

In Mexico, *A*. *ovale* ticks have been collected from 8 species of mammals in 10 of 32 states (*2*). Despite the wide distribution of *A*. *ovale* ticks in Mexico, attempts to identify *R*. *parkeri* strain Atlantic Rainforest in this species are lacking.

During July–August 2018, we collected *A*. *ovale* ticks from dogs in 3 municipalities, Alvarado (18°46′52′′N, 95°45′26′′W), Catemaco (18°30′36.30′′N, 95°02′08.61′′W), and Martínez de la Torre (20°04′00′′N, 97°03′00′′W), in the state of Veracruz, Mexico (Figure, panel A). Ticks were harvested from owned dogs during their evaluations at veterinary clinics and from free-roaming dogs during vaccination campaigns conducted by local rabies vaccination programs. We identified ticks morphologically using a standard taxonomic key (*2*), fixed them in absolute ethanol, and stored them at 4°C.

**Figure:**
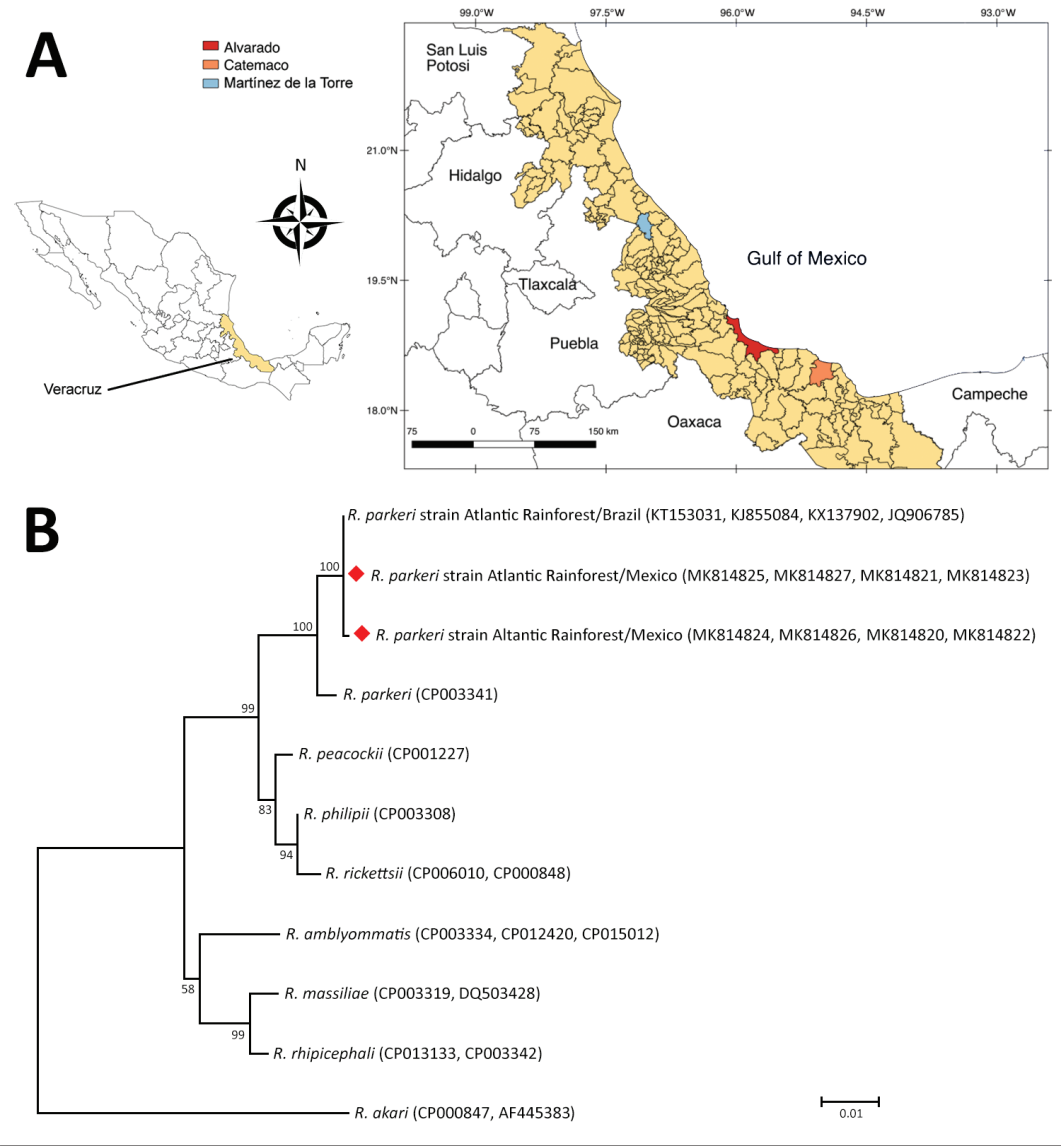
*Amblyomma ovale* tick sampling sites and phylogenetic analysis of tickborne *Rickettsia parkeri* strain Atlantic Rainforest isolates (diamonds), state of Veracruz, Mexico, July–August 2018. A) Sites where *A. ovale* ticks were collected from dogs to assess prevalence of *R*. *parkeri* strain Atlantic Rainforest. Inset shows location of Veracruz state in Mexico. QGis (https://www.qgis.org) was used for map construction. B) Maximum-likelihood phylogenetic tree generated with concatenated segments of the *gltA*, *htrA*, *sca0*, and *sca5* genes (2,476 bp total) of several members of spotted fever group *Rickettsia*. Bootstrap values >50 are indicated at nodes. GenBank accession numbers are provided. Scale bar indicates nucleotide substitutions per site.

To extract DNA, we used the Cheelex-100 protocol as previously reported (*7*,*8*). To evaluate the DNA quality of samples, we amplified a 400-bp segment of the ixodid 16S rRNA gene (*5*). We screened DNA extracts for *Rickettsia* species using a PCR targeting an 800-bp segment of the citrate synthase (*gltA*) gene. With *gltA*-positive samples, we performed PCRs amplifying segments of the *htrA* (549-bp), *sca0* (532-bp), and *sca5* (862-bp) genes (*7*,*8*). We purified PCR products using Agencourt AMPure XP (https://www.beckman.com) and sequenced amplicons on the ABI 3730xL DNA Analyzer (https://www.thermofisher.com) at the Sequencing Unit of the National Institute of Genomic Medicine (Mexico City, Mexico). We generated consensus sequences using Geneious 2019.1.3 (https://www.geneious.com) and compared these sequences with those of validated *Rickettsia* species deposited in GenBank using the blastn tool (https://blast.ncbi.nlm.nih.gov). We performed global alignments using ClustalW (http://www.clustal.org), concatenated sequences in BioEdit (https://bioedit.org), and then constructed phylogenetic trees in MEGA 6.0 (https://megasoftware.net) using the maximum-likelihood method and 10,000 bootstrap replicates.

We collected 22 adult (16 female, 6 male) *A*. *ovale* ticks from 6 dogs (tick density of 2–5 ticks per dog). We could amplify ixodid 16S sequences from all samples. We sequenced the 16S gene of 1 female (GenBank accession no. MK792953) and 1 male tick, and both exhibited 99.5% (404/406 bp) sequence identity with sequences of *A*. *ovale* ticks from Colombia (GenBank accession nos. MF353104.1–5.1). Six (27.3%) specimens tested positive for *Rickettsia* DNA, including 1 female specimen from Alvarado, 2 female specimens from Martínez de la Torre, and 2 female specimens and 1 male specimen from Catemaco. The *gltA*, *htrA*, *sca0*, and *sca5* gene segments could be amplified for all 6 samples. Each gene segment was 99%–100% identical to that of the *R*. *parkeri* strain Atlantic Rainforest from Brazil and Argentina (Figure, panel B; data not shown). Phylogenetic analysis corroborated the presence of 2 *R*. *parkeri* strain Atlantic Rainforest haplotypes: 1 for the northern region (Martínez de la Torre; GenBank accession nos. MK844821, MK844823, MK844825, MK844827) and 1 for the central and southern regions (Alvarado and Catemaco; GenBank accession nos. MK844820, MK844822, MK844824, MK844826) of Veracruz. With a bootstrap value of 100, both haplotypes clustered in a clade comprising other *R*. *parkeri* strains.

Our findings document *R*. *parkeri* strain Atlantic Rainforest farther north than previous reports (*4*–*6*). The discovery of this pathogen in ticks associated with dogs in different localities of Veracruz has implications for public health safety. In this state, the Ministry of Health reported 22 cases of spotted fever during 2015–2017 (*9*). *R. rickettsii*, the etiologic agent of Rocky Mountain spotted fever, has been previously described in *A*. *mixtum* (formerly *A*. *cajennense*) ticks collected from Veracruz (*10*), suggesting the potential for co-circulation of *R*. *rickettsii* and *R*. *parkeri* in ticks in this state. Two other *R*. *parkeri* lineages have been detected circulating in Mexico: *R*. *parkeri* strain black gap in the rabbit tick (*Dermacentor parumapertus*) in Sonora and Chihuahua (*7*) and *R*. *parkeri* sensu stricto associated with *A*. *maculatum* ticks (*8*). These findings emphasize the need for enhanced surveillance studies of these rickettsia in Mexico to better elucidate the evolutionary, ecologic, and public health relevance of the various *R*. *parkeri* strains.
